# Association between thyroid dysfunction, metabolic disturbances, and clinical symptoms in first-episode, untreated Chinese patients with major depressive disorder: Undirected and Bayesian network analyses

**DOI:** 10.3389/fendo.2023.1138233

**Published:** 2023-02-28

**Authors:** Pu Peng, Qianjin Wang, Xiao E Lang, Tieqiao Liu, Xiang-Yang Zhang

**Affiliations:** ^1^ Department of Psychiatry, National Clinical Research Center for Mental Disorders, The Second Xiangya Hospital of Central South University, Changsha, Hunan, China; ^2^ Department of Psychiatry, First Hospital of Shanxi Medical University, Taiyuan, China; ^3^ CAS Key Laboratory of Mental Health, Institute of Psychology, Chinese Academy of Sciences, Beijing, China; ^4^ Department of Psychology, University of Chinese Academy of Sciences, Beijing, China

**Keywords:** major depressive disorder, network analysis, metabolic disturbances, thyroid dysfunction, anxiety, psychotic symptoms

## Abstract

**Aims:**

Thyroid dysfunction and metabolic disturbances are common in major depressive disorder (MDD) patients. We aimed to assess the relationship between thyroid dysfunction, metabolic disturbances, and clinical symptoms in Chinese first-episode, drug-naïve (FEDN) MDD patients using undirected and Bayesian network methods.

**Methods:**

1718 FEDN MDD patients were recruited. Serum levels of free triiodothyronine (FT3), free thyroxine (FT4), thyroid stimulating hormone (TSH), anti-thyroglobulin (TgAb), thyroid peroxidases antibody (TPOAb), total cholesterol (TC), total triglycerides (TG), high-density lipoprotein (HDL-C), low-density lipoprotein (LDL-C), and glucose were assessed. Blood pressure and body mass index were measured. Hamilton Rating Scale for Depression (HAMD), Hamilton Rating Scale for Anxiety, and positive subscale of Positive And Negative Syndrome Scales were used to detect clinical symptoms. An undirected network with EBICglasso default and a directed acyclic graph (DAG) using the Bayesian network approach was conducted.

**Results:**

The prevalence rates of clinical symptoms, thyroid dysfunction, and metabolic dysfunction were as follows: anxiety (n=894, 52%), psychotic symptoms (171, 10%), subclinical hypothyroidism (SCH, n=1041, 61%), abnormal TgAb (n=297, 17%), abnormal TPOAb (n=438, 25%), hyperthyroidism (n=5, 0.3%), hypothyroidism (n=3, 0.2%), hyperglycemia (n=241, 14%), hypertriglyceridemia (n=668, 39%), low HDL-C (n=429, 25%), hypercholesterolemia (421, 25%), abnormal TC (357, 21%), abnormal LDL-C (185, 11%). overweight or obesity (n=1026, 60%), and hypertension (n=92, 5.4%). Both networks demonstrated serum TSH and TC levels and the severity of depression played an important role in the pathophysiology of MDD.

**Conclusions:**

MDD patients may have thyroid and metabolic dysfunction in the early stage. Targeting hypercholesterolemia, depressive symptoms, and SCH in MDD patients may hold promise in reducing clinical symptoms, metabolic disturbances, and thyroid dysfunction.

## 1 Introduction

Major depressive disorder (MDD) is the most common and severe psychiatric disorder, which leads to a significant impact on quality of life and functioning (1, 2). It is the leading cause of disability-adjusted life years in adults and affects approximately 300 million people worldwide (3). Emerging studies have demonstrated biological changes in MDD patients, such as impaired glucose and lipid metabolism, thyroid dysfunction, and obesity (4–6). Previous studies have found that metabolic disorders and/or thyroid dysfunction increase the risk of MDD (4, 7, 8). More importantly, a growing number of studies have shown that metabolic disorders and thyroid dysfunction are positively associated with poor response to antidepressants (9), a higher risk of readmission (10), and more severe comorbidities such as suicide (11) and anxiety (12). Taken together, these findings highlight the important role of metabolic disorders and thyroid dysfunction in the pathophysiology of MDD.

Despite the great interest in the abnormalities of metabolism and thyroid function in MDD patients, there are some important limitations of the current evidence. First, most prior studies have had relatively limited sample sizes and have ignored potential confounding factors such as antidepressants and disease courses (13, 14). Second, although studies in the general population have demonstrated substantial associations between metabolic disorders and thyroid dysfunction (15–19), they are most often studied alone in patients with MDD. Clarifying whether and how metabolic disorders, thyroid disorders, and clinical symptoms reinforce and interact with each other in patients with MDD will deepen our understanding of the pathophysiology of MDD.

Network analysis is a promising tool for understanding the associations between variables (20, 21). In the network model, each variable is represented as a “node”. The edges represent the unique association between two variables after adjusting for the rest of the variables within the network. Through network analysis, we can estimate the interaction between thyroid dysfunction, metabolic disturbance, and clinical symptoms in MDD patients in more detail. It allows us to identify the most influential variables in the network, which may serve as the target for clinical interventions (20).

To date, network approaches have been used to identify associations between pro-inflammatory proteins, lipid markers, genetic factors, and depressive symptoms in patients with MDD (22, 23). However, no previous studies have assessed networks of thyroid dysfunction, metabolic disorders, and clinical symptoms in patients with MDD. To fill this gap, we conducted the present study. We recruited a large sample of first-episode drug-naïve (FEDN) patients with MDD, which minimized the impact of medical therapy, disease duration, and comorbidities. We conducted two networks. The first network was undirected and was used to explore the most relevant connections and core variables in the thyroid-metabolism-clinical network. For the second network, we performed a Bayesian network analysis to obtain directions of potential causal relationships between thyroid dysfunction, metabolic disorders, and clinical symptoms.

## 2 Materials and methods

### 2.1 Study setting and procedure

FEDN MDD outpatients were recruited during 2015-2017 at the Department of Psychiatry, First Hospital, Shanxi Medical University. Participants should meet the following criteria: (1) diagnosis of MDD by two independently trained clinical psychiatrists according to the Structured Clinical Interview for DSM-IV (SCID); (2) 17-item Hamilton Rating Scale for Depression (HAMD) total score ≥ 24; (3) age between 18-60 years old and Han Chinese; (4) depressive symptoms were first-episode without any prior medications, including antidepressant, antipsychotic, or thyroid treatment medication; and (4) MDD of disease duration was no more than 24 months.

Exclusion criteria included: (1) the presence of any other major DSM-IV Axis I disorder based on SCID; (2) the presence of serious physical illness, such as organic brain disease or severe infection; (3) any substance abuse or dependence other than tobacco; (3) pregnant or lactating women; and (4) refusal to give informed consent.

The work described has been carried out in accordance with The Code of Ethics of the World Medical Association (Declaration of Helsinki). The study was approved by the Institutional Review Board (IRB) of the First Hospital of Shanxi Medical University (No. 2016-Y27). All participants gave their written informed consent.

### 2.2 Measurements

#### 2.2.1 Basic information

The demographic information including gender, age, married status, education level, age at first episode onset, and duration of MDD courses were collected through self-administrated questionnaires.

#### 2.2.2 Clinical assessments

Two trained psychiatrists independently administrated the SCID to each participant. They assessed depression, anxiety, and psychotic symptoms using the Chinese version of the HAMD, the Hamilton Anxiety Rating Scale (HAMA), and the Positive and Negative Symptom Scale (PANSS) positive subscale, respectively (24, 25). Following the previous study (26), patients were divided into the without or possible anxiety group (HAMA scores 0-13), mild to moderate anxiety group (HAMA scores 14-20), significant anxiety group (HAMA scores 21-28), and severe anxiety group (HAMA scores >28) based on the HAMA scores. A cutoff point of 15 was used to determine the presence of psychotic symptoms (27). The inter-observer correlation coefficients of the scores on the three scales were >0.8.

#### 2.2.3 Thyroid function and metabolic parameters

Before participants received any medication, their fasting blood samples were collected between 6 am and 8 am. Serum levels of the following biochemical parameters were assessed: free triiodothyronine (FT3), free thyroxine (FT4), thyroid stimulating hormone (TSH), anti-thyroglobulin (TgAb), thyroid peroxidases antibody (TPOAb), total cholesterol (TC), total triglycerides (TG), high-density lipoprotein (HDL-C), low-density lipoprotein (LDL-C), and fasting glucose. Thyroid hormones (TSH, TPOAb, TgAb, FT3, and FT4) were measured on a Roche C6000 Electrochemiluminescence Immunoassay Analyzer (Roche Diagnostics, Indianapolis, IN, USA), while metabolic parameters (HDL-C, LDL-C, TC, TG, and glucose) were assessed on a Cobas E610 (Roche, Basel, Switzerland) in the laboratory of Shanxi Medical University. The blood pressure, height, and weight were taken by trained nurses. Body mass index (BMI) was calculated with the equation: BMI= Weight (kg)/Height (m)^2^. The definition of metabolic disturbances and thyroid dysfunction were as follows: (1) overweight or obesity: BMI≥24; (2) hyperglycemia: glucose≥6.1mmol/L; (3) hypertension: SBP≥140 mmHg and/or DBP≥90mmHg; (4) hypertriglyceridemia: TG≥2.3 mmol/L; (5) low HDL: HDL-C ≤ 1.0 mmol/L; (6) hypercholesterolemia: TC≥6.2 mmol/L or LDL-C≥4.1 mmol/L; (7)abnormal TgAb: TgAb≥115 IU/L; (8) abnormal TPOAb: TPOAb ≥34 IU/L; (9) subclinical hypothyroidism (SCH): TSH >4.2 mIU/L with a normal fT4 concentration (10–23 pmol/L); (10) hyperthyroidism: TSH<0.27 mIU/L and FT4 over 23 pmol/L, and (11) hypothyroidism: TSH >4.2 mIU/L with a low FT4 concentration (<10 pmol/L).

### 2.3 Statistical analysis

According to the Shapiro-Wilk test, the continuous variables in this study were not normally distributed. Therefore, we denoted continuous variables as the median and interquartile range (IRQ; 25-75%) and categorical variables as frequencies and percentages. All statistical analyses were performed on R(ver.4.20). We conducted the partial correlation test to evaluate the association between clinical symptoms (HAMA, HAMD, PANSS), thyroid hormones (TPOAb, TgAb, TSH, FT3, FT4), and metabolic parameters (HDL-C, LDL-C, TC, TG, glucose, BMI, SBP, and DBP). Basic information including age, age at first episode onset, illness duration, gender, education, and married status were controlled as covariates.

### 2.4 Undirected network analysis

Thyroid hormones, metabolic parameters, and clinical symptoms were included in the network analyses. Following previous studies, we converted the data set to normal using a non-normal transformation through the R package “huge” (28). The network was estimated using the EBICglasso model, which is the most widely used model in psychopathology networks (29, 30). The R packages “qgraph” and “bootnet” were applied for the visualization of the network. In the network, each “node” represented one item of thyroid hormones, metabolic parameters, and clinical information. After adjusting the other nodes in the network, the unique connection between two nodes is visualized as an “edge” between the nodes. The thickness and color of the edges indicate the strength and direction of the relationship. The thicker the edge, the stronger the association. Red and blue edges represent negative and positive associations, respectively.

We calculate the centrality index “strength” to identify the central nodes in the network. The strength is the sum of the absolute edge weights that directly connect a node to the other nodes in the network. Nodes with the highest strength are considered central nodes, and they have the strongest influence on other variables in the network. Central nodes are important in the creation of networks (31–33) and can be used as potential targets for clinical interventions. We also assessed the “predictability” of each node by Rpackage “MGM”. Similar to the adjusted R^2^ in regression models, the predictability of a node indicates the extent to which the variance of a node can be predicted by other nodes in the network (34). Nodes with high predictability might be easily changed by changing their related nodes.

The case-dropping procedure was applied to evaluate the stability of our network. The correlation stability coefficient (CS-C) of node strength was calculated, with CS-C above 0.5 implying high stability. To test the accuracy of edges estimated within the network, we conducted the nonparametric bootstrapping with 1000 bootstrap samples.

We used a case-dropping procedure to assess the stability of our network. The correlation stability coefficient (CS-C) of the node strength was calculated, and a CS-C above 0.5 indicated high stability. To test the accuracy of the estimated edges within the network, we performed nonparametric bootstrapping with 1000 bootstrap samples.

We further compared our networks with the Rpackage “Network Comparison Tool”. Three subgroups were analyzed including gender (female versus male), illness duration (≤5 months versus >5 months), and age (18-45 years old versus 46-60 years old). The overall strength (absolute sum value of all edge weights) and network structure (distribution of edge weights) were evaluated between subgroups of networks.

### 2.5 Bayesian network analysis

Finally, we performed directed acyclic graphs (DAG) to assess the putative direction of causal relationships between thyroid dysfunction, metabolic disorders, and clinical symptoms. DAG is an emerging method for network analysis. It allows us to detect and represent the most likely direction of causality between variables based on the conditional dependence between each pair of variables in the presence of other variables in the network (35, 36). More importantly, studies have shown that variables at the top of the DAG may have a higher predictive priority and more salience and should receive higher priority in treatment (37, 38). Following previous studies (36, 39), we performed DAG by the R package “bnlearn”. A hill-climbing algorithm was chosen to estimate the network. According to the protocol of Scutari M & Nagarajan R (40), the optimal cut point method was used to retain edges with high sensitivity and specificity.

## 3 Result

### 3.1 Sample characteristics

A total of 1718 patients (female: 1130, male: 588) with FEDN MDD were recruited (**Table 1**). The median age at the first episode onset was 34 (23, 41) years. 43% (n=740), 32% (n=549), and 25% (n=429) of the participants were between 18-30, 31-45, and 46-60 years old, respectively. 171 (10%) suffered from psychotic symptoms. According to HAMA scores, the participants could be divided into without or possible anxiety (n=1, 0.1%), mild to moderate anxiety (n=853, 47.9%), significant anxiety (n=849, 49.4%), and severe anxiety (n=45, 2.6%). The prevalence of thyroid dysfunction and metabolic disorders was as follows: SCH (n=1041, 61%), TgAb abnormalities (n=297, 17%), TPOAb abnormalities (n=438, 25%), hyperthyroidism (n=5, 0.3%), hypothyroidism (n=3, 0. 2%), hyperglycemia (n=241, 14%), hypertriglyceridemia (n=668, 39%)), low HDL-C (n=429, 25%), hypercholesterolemia (421, 25%), abnormal TC (357, 21%), abnormal LDL-C (185, 11%), overweight or obese (n=1026, 60%) and hypertensive (n=92, 5.4%).

**Table 1:** Sample characteristics.

Variable	Overall, N = 1,718^1^
**Age, year**	34 (23, 45)
Age groups	
18-30	740 (43%)
31-45	549 (32%)
46-60	423 (25%)
**Duration, month**	5 (3, 8)
**Age at first episode onset, year**	34 (23, 45)
**Gender**	
Male	588 (34%)
Female	1,130 (66%)
Education	
Below college	1,173 (68%)
College or above	545 (32%)
**Married**	1,216 (71%)
PANSS	7 (7, 7.8)
**Psychotic symptom**	171 (10.0%)
**HAMD**	30 (28, 32)
**HAMA**	21 (18, 23)
Anxiety	
Without or possible anxiety	1 (0.1%)
Mild to moderate anxiety	853 (47.9%)
Significant anxiety	849 (49.4%)
Severe anxiety	45 (2.6%)
**TSH, uIU/L**	4.91 (3.11, 6.66)
**TgAb, IU/L**	21 (14, 44)
**TPOAb, IU/L**	17 (12, 35)
**FT3, pmol/L**	4.92 (4.38, 5.41)
**FT4, pmol/L**	16.5 (14.4, 18.7)
**Glucose, mmol/L**	5.34 (4.94, 5.80)
**TC, mmol/L**	5.22 (4.46, 6.00)
**HDLC, mmol/L**	1.23 (1.01, 1.42)
**TG, mmol/L**	1.97 (1.40, 2.77)
**LDLC, mmol/L**	2.96 (2.38, 3.52)
**BMI, kg/m2**	24.23 (23.22, 25.60)
**SBP, mmHg**	120 (112, 127)
**DBP, mmHg**	76 (70, 80)
**Abnormal TgAb**	297 (17%)
**Abnormal TPOAb**	438 (25%)
**SCH**	1,041 (61%)
**Hyperthyroidism**	5 (0.3%)
**Hypothyroidism**	3 (0.2%)
**Hyperglycemia**	241 (14%)
**Low HDL**	429 (25%)
**Overweight or obesity**	1,026 (60%)
**Hypertriglyceridemia**	668 (39%)
**Abnormal TC**	357 (21%)
**Abnormal LDL-C**	185 (11%)
**Hypertension**	92 (5.4%)
**Hypercholesterolemia**	421 (25%)

^1^Median (IQR); n (%). SCH, subclinical hypothyroidism; HAMD, Hamilton Depression Rating Scale; HAMA, Hamilton Anxiety Rating Scale; PANSS, the Positive and Negative Syndrome Scale; TSH, thyroid-stimulating hormone; FT3, free triiodothyronine; FT4, free thyroxine; TgAb, antithyroglobulin; TPOAb, thyroid peroxidases antibody; TC, total cholesterol; HDL-C, high-density lipoprotein; LDL-C, low-density lipoprotein; TG, total triglycerides; BMI, body mass index.

### 3.2 Correlation between thyroid dysfunction, metabolic disturbances, and clinical symptoms

We found that clinical symptoms, metabolic disturbances, and thyroid dysfunction were strongly correlated after adjusting for basic information (**Table S1**). Specifically, the partial correlation test demonstrated substantial inter-relationships between three clinical symptoms: HAMA-HAMD (r=0.615), HAMD-PANSS (r=0.539), and HAMA-PANSS (r=0.610) (all p<0.001). Notably, TSH levels were strongly and positively associated with HAMD (r=0.466), HAMA (r=0.344), PANSS (r=0.367), glucose (r=0.444), TC (r=0.545), SBP (r=0.551), and DBP (r=0.356) (all p<0.001). We also found a solid positive relationship between TC and HAMD (r=0.553, p<0.001).

### 3.3 Undirected network of thyroid dysfunction, metabolic disturbance, and clinical symptoms in FEDN MDD patients

**Figure 1:**
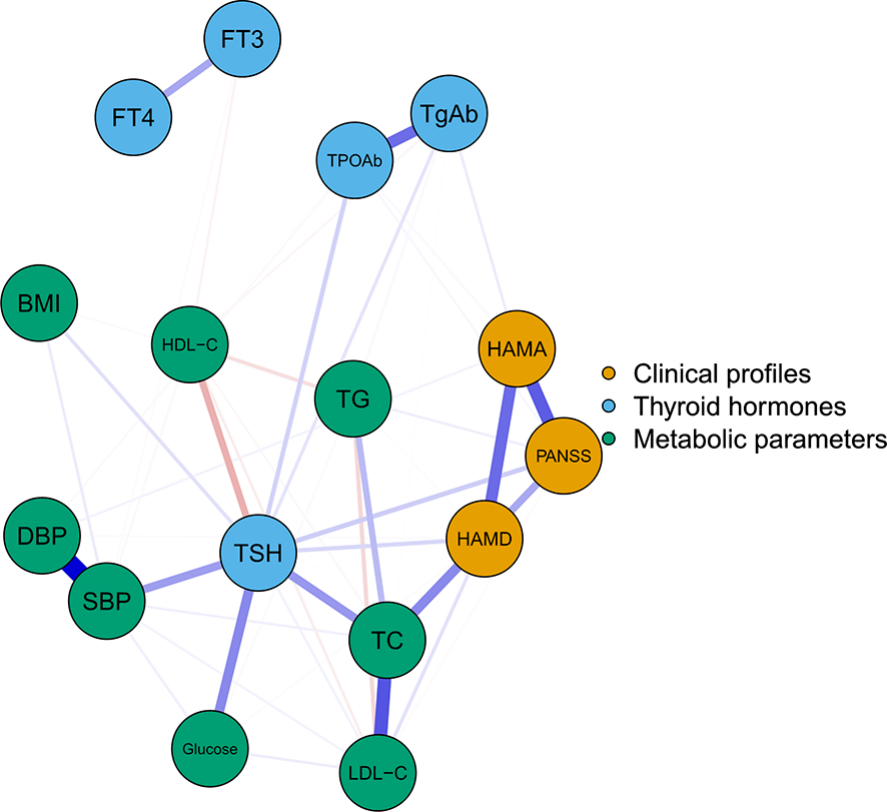
The undirected network of thyroid hormones, metabolic parameters, and clinical symptoms The blue, green, and orange nodes represent thyroid hormones, metabolic parameters, and clinical profiles, respectively. Blue edges indicate positive association while red edges indicate negative association. Thicker edge implies a stronger association. HAMD, Hamilton Depression Rating Scale; HAMA, Hamilton Anxiety Rating Scale; PANSS, the Positive and Negative Syndrome Scale; TSH, thyroid-stimulating hormone; FT3, free triiodothyronine; FT4, free thyroxine; TgAb, antithyroglobulin; TPOAb, thyroid peroxidases antibody; TC, total cholesterol; HDL-C, high-density lipoprotein; LDL-C, low-density lipoprotein; TG, total triglycerides; BMI, body mass index.

**Figure 1** illustrates the network of thyroid dysfunction, metabolic disorders, and clinical symptoms in patients with FEND MDD. The network consists of 16 nodes with a density of 0.44 (53/120). Visually, the node TSH is located in the center of the network. It shows a strong relationship with metabolic disorders (i.e. SBP, Glucose, and TC). It was also positively correlated with HAMA, HAMD, and PANSS. We also observed a strong relationship between HAMA, HAMD, and PANSS. The ten strongest edges in the network were SBP-DBP, followed by TPOAb-TgAb, TC-LDL-C, HAMD-PANSS, HAMA-PANSS, TSH-Glucose, HAMD-HAMA, HAMD-TC, TSH-TC, and TSH-TC. According to the results of the nonparametric bootstrap procedure, these edges were statistically stronger than the other edges within the network (**Figure S1**). The value of the edges is listed in **Table S2**.

**Figure 2:**
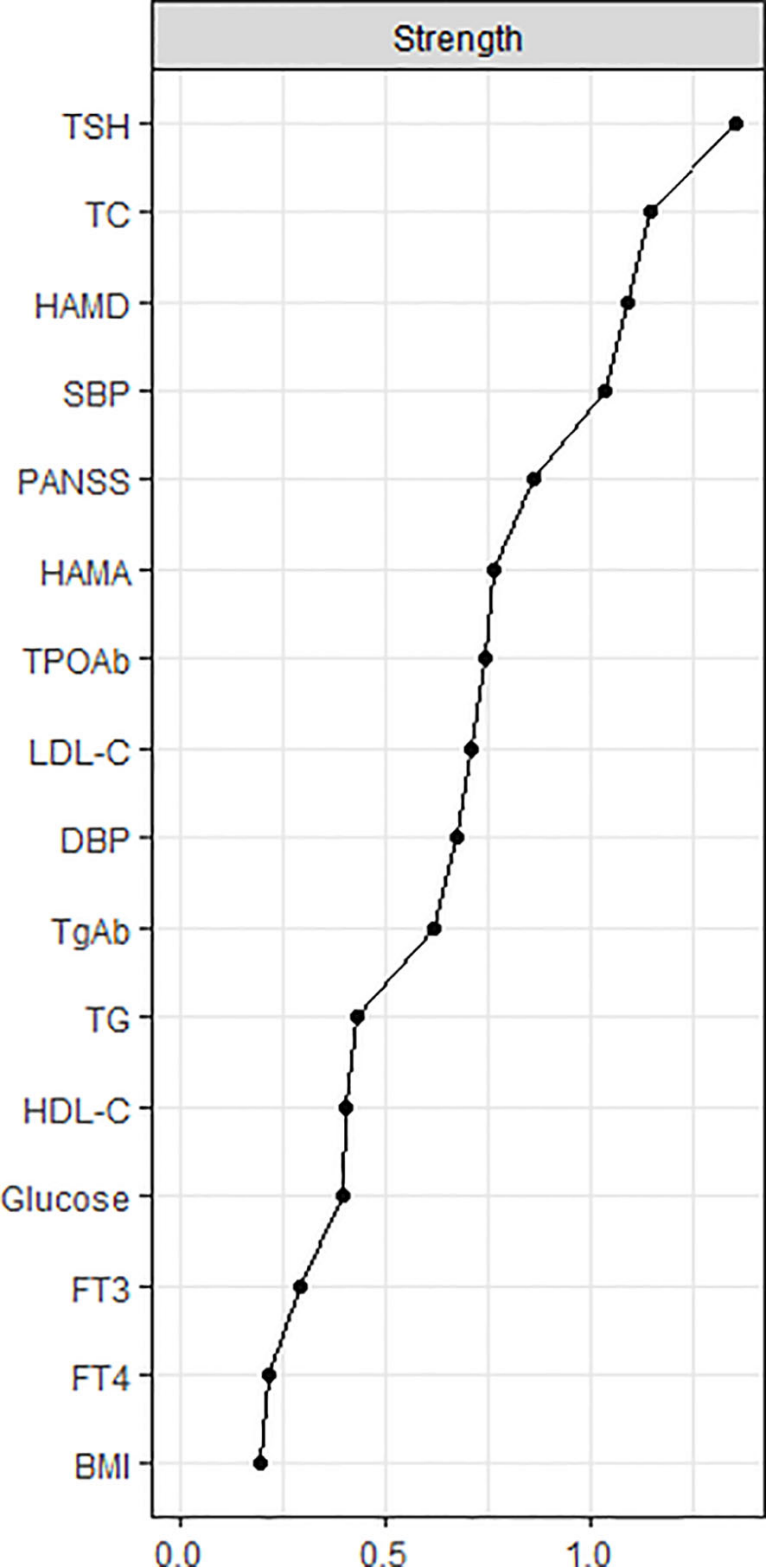
The central nodes of the network. The X-rays represented the strength of each node. Nodes with higher strength have stronger impact in other nodes within the network. HAMD, Hamilton Depression Rating Scale; HAMA, Hamilton Anxiety Rating Scale; PANSS, the Positive and Negative Syndrome Scale; TSH, thyroid-stimulating hormone; FT3, free triiodothyronine; FT4, free thyroxine; TgAb, antithyroglobulin; TPOAb, thyroid peroxidases antibody; TC, total cholesterol; HDL-C, high-density lipoprotein; LDL-C, low-density lipoprotein; TG, total triglycerides; BMI, body mass index.

The centrality plot (**Figure 2**) shows that TSH, TC, and HAMD scores have the highest strength, implying that they are the central nodes in the network. They have a statistically higher strength than the other nodes (**Figure S2**). The predictability of the nodes within the network is shown in **Table S3**. Notably, the predictability of HAMD was 0.604, HAMA was 0.456, and PANSS was 0.516, indicating that half of the severity of depression, anxiety, and psychotic symptoms could be explained by the other nodes in the network.

The network had good stability and accuracy (**Figures S3, S4**). The CS-C was 0.75, indicating that the network was highly correlated with the original network even after discarding 75% of the original data (r=0.7).

### 3.4 Network comparison test

We compared the networks of thyroid dysfunction, metabolic disorders, and clinical symptoms according to gender, age, and illness duration (**Figure S5**). We did not observe any differences in the overall strength and network structure of the networks between the three subgroups.

### 3.5 Bayesian network analysis

**Figure 3:**
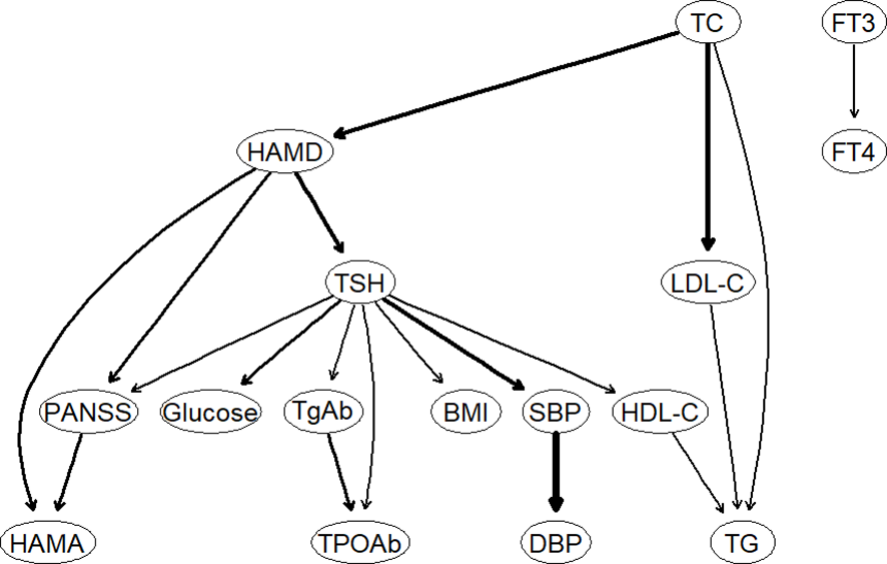
The Bayesian network of thyroid hormones, metabolic parameters, and clinical symptoms. HAMD, Hamilton Depression Rating Scale; HAMA, Hamilton Anxiety Rating Scale; PANSS, the Positive and Negative Syndrome Scale; TSH, thyroid-stimulating hormone; FT3, free triiodothyronine; FT4, free thyroxine; TgAb, antithyroglobulin; TPOAb, thyroid peroxidases antibody; TC, total cholesterol; HDL-C, high-density lipoprotein; LDL-C, low-density lipoprotein; TG, total triglycerides; BMI, body mass index.

**Figure 3** illustrates the DAG of thyroid disorders, metabolic disorders, and clinical symptoms in patients with FEDN MDD. Consistent with the undirected network analysis, HAMD, TSH, and TC were located at the top of the DAG, indicating that they may trigger and maintain metabolic disorders, thyroid dysfunction, and clinical symptoms in patients with FEDN MDD. Similarly, we found that TSH was closely associated with metabolic disorders. It was upstream of glucose disturbance, hypertension, obesity, and HDL-C. The DAG also suggests that TSH may contribute to more severe psychotic symptoms.

## 4 Discussion

To our knowledge, this is the first study to describe the relationship between thyroid dysfunction, metabolic disorders, and clinical symptoms in a large sample of FEDN MDD patients by a network approach. Our study highlights the common metabolic burden and thyroid dysfunction in patients with MDD. Both the undirected network and DAG showed a dominant role of TC, TSH, and depression severity in patients with MDD. Therefore, targeting abnormal TSH, TC, and depressive symptoms may hold great promise for reducing MDD-related thyroid dysfunction, metabolic disturbances, and clinical symptoms.

Our study suggested high TSH levels (i.e., SCH) played a vital role in triggering metabolic disturbances and clinical symptoms in FEDN MDD patients. In our study, the prevalence of SCH in our sample was 61%, much higher than that in the Chinese general population (range: 3.4%-12.93%) (42–44). More importantly, we found that TSH levels were highly correlated with the severity of depressive symptoms in FEDN MDD patients. The shared biological mechanisms between MDD and SCH, such as disturbances in the hypothalamic–pituitary–adrenal axis and changes in the hormone levels including somatostatin and serotonin might contribute to their tight associations (45). In recent years, studies also indicated that the Wnt/β-catenin pathway might play a role in the association between SCH and depression. Interestingly, the DAG indicated that the TSH levels might be the outcome rather than a cause of depressive symptoms in MDD. However, there were very few, if any, longitudinal studies that evaluated whether depressive symptoms could predict increased TSH levels. Further studies are needed to validate our findings.

In addition to depressive symptoms, we demonstrated a strong positive correlation between TSH levels and psychotic symptoms in patients with FEDN MDD. To date, only a few studies have evaluated their relationship and have yielded inconsistent results. For example, Liu et al. found that serum TSH levels were independently associated with psychotic symptoms in 1279 patients with MDD (11). Another large study of 1410 patients with MDD showed that hypothyroidism was positively associated with psychotic symptoms (41). However, Contreras et al. found no difference in TSH blunting in MDD patients with and without psychotic symptoms (46). A possible explanation might be the complicated medication in the treatment of MDD and the varied sample size, which might impact the association between psychotic symptoms and TSH levels. Furthermore, most of the current evidence on the association between TSH and psychosis came from the observational cross-sectional study. The underlying mechanism is still largely unexplored. Hence, further studies were still needed to confirm our findings.

Consistent with previous studies in the general population (18, 47), high TSH levels exhibited substantial associations with impaired metabolism, including hypertension, hyperglycemia, obesity, and hypercholesterolemia. To date, only a few studies have investigated their relationship in the context of MDD (48, 49), and these studies reported similar results to ours. For example, Kim et al. found that SCH increased the risk of metabolic syndrome (MetS) approximately 7-fold in Korean adults with depression (48). Zhao et al. found that SCH was independently associated with BMI, TC, and LDL-C in MDD patients (49). A few possible explanations might account for the high co-occurrence of SCH and metabolic disturbances. First, studies have suggested that high TSH levels could directly impact lipid metabolism by binding the TSH receptor (50). High TSH levels could promote cholesterol synthesis and inhibit cholesterol clearance, which is independent of FT3 and FT4 (50). Second, recent studies suggested that hepatic endoplasmic reticulum stress induced by SCH played an important role in dyslipidemia and impaired glucose metabolism (51, 52). Interestingly, a strong association between SCH and metabolic disorders has been reported in patients with other psychiatric disorders. For example, a recent study showed higher serum TSH levels in schizophrenia patients with comorbid MetS (53). Taken together, the co-occurrence of SCH and metabolic disorders may be common in psychiatric patients, which calls for regular screening of this population.

Further, we found that high TC levels were another important factor in the network of thyroid dysfunction, clinical symptoms, and metabolic disorders in patients with FEDN MDD. Notably, it was highly correlated with the severity of depression in this study, which is consistent with some previous studies (54–56). There are several explanations for their strong association. First, MDD patients with more severe depressive symptoms may have a more irregular lifestyle, such as late nights and an unhealthy diet, which leads to increased TC levels. Second, inflammation may act as a bridge between hypercholesterolemia and MDD. Hypercholesterolemia has been found to trigger the activation of the NLRP3 inflammasome, which leads to a chronic state of inflammation (57). A growing number of studies suggest a bidirectional relationship between MDD and inflammation (58, 59). However, we did not collect the variables associated with inflammation. Therefore, further studies are needed to test our hypothesis.

The present study has several important clinical implications. First, our study highlights the heavy metabolic burden and thyroid dysfunction in patients with FEDN MDD, which calls for regular screening of this population. Second, we found that greater depressive symptoms, higher TSH levels, and higher TC levels may play an important role in metabolic disturbances, thyroid dysfunction, and clinical symptoms, which should be given higher priority in treatment. Several clinical trials have demonstrated the potential effectiveness of anti-SCH and anti-hypercholesterolemia in the treatment of MDD (60–62), which supported our findings. For example, several studies have demonstrated the efficacy and safety of statins (one of the most widely used drugs for the treatment of hypercholesterolemia) in MDD (60, 61). A recent review suggests that thyroid hormone therapy may be a promising strategy for refractory MDD (62). Thyroid hormone therapy was also found to improve lipid metabolism in SCH patients (63). However, it should be noted that the results of thyroid hormone therapy in the treatment of MDD are inconsistent (64, 65), which warranted further studies.

The study has the following limitations. First, it should be acknowledged that the study was observational and cross-sectional, which inhibited us from drawing a causal relationship between clinical symptoms, metabolic disturbances, and thyroid dysfunction among patients with MDD. Second, despite the large sample size, this study was conducted in a single hospital. Whether our findings can be generalized to other populations remains unclear. Third, we did not control for several important potential confounders, such as diet, smoking, drinking, and pro-inflammatory factors. Therefore, further longitudinal studies which provide a more comprehensive assessment of lifestyle factors are needed to validate our findings.

In summary, this study described the association between thyroid dysfunction, metabolic disorders, and clinical symptoms in a large sample of FEDN MDD patients by both undirected and Bayesian network approaches. Both networks suggest that TSH, TC, and depression severity are of high importance in patients with MDD and should be treated with high priority.

## Data availability statement

The raw data supporting the conclusions of this article will be made available by the authors, without undue reservation.

## Ethics statement

The studies involving human participants were reviewed and approved by Institutional Review Board (IRB) of the First Hospital of Shanxi Medical University. The patients/participants provided their written informed consent to participate in this study.

## Author contributions

PP: Formal analysis, Writing - original draft. QW: Writing – review & editing. XL: Writing – review & editing. TL: Conceptualization, Writing–review & editing. X-YZ: Conceptualization, Writing–review & editing. All authors contributed to the article and approved the submitted version.
