# Supplementary material for: Association between thyroid dysfunction, metabolic disturbances, and clinical symptoms in first-episode, untreated Chinese patients with major depressive disorder: Undirected and Bayesian network analyses
Source: Front Endocrinol (Lausanne). 2023 Feb 28;14:1138233. doi: 10.3389/fendo.2023.1138233 (PMC10013149; doi:10.3389/fendo.2023.1138233)
Supplement: Supplementary file 1 [file DataSheet_1.docx]

Supplementary materials for “Association between thyroid dysfunction, metabolic disturbances, and clinical symptoms in first-episode, untreated Chinese patients with major depressive disorder: undirected and Bayesian network analyses”

Table S1 Partial correlation matrix of thyroid hormones, metabolic parameters, and clinical symptoms

Table S2 The edge between thyroid hormones, metabolic parameters, and clinical symptoms

Table S3 Predictability of nodes in the network

Figure S1 Estimation of edge difference within the thyroid hormones-metabolic parameters-clinical symptoms network by bootstrapped difference test.

Figure S2 Estimation of node strength difference within the thyroid hormones-metabolic parameters-clinical symptoms network by bootstrapped difference test.

Figure S3 The stability of the network

Figure S4 The accuracy of the network

Figure S5 Network comparison by gender, age, and illness duration

Table S1 Partial correlation matrix of thyroid hormones, metabolic parameters, and clinical symptoms

Table S2 The edge between thyroid hormones, metabolic parameters, and clinical symptoms

Table S3 Predictability of nodes in the network

| Variable | Predictability |
| --- | --- |
| HAMD | 0.604 |
| HAMA | 0.456 |
| PANSS | 0.516 |
| TSH | 0.52 |
| TgAb | 0.298 |
| TPOAb | 0.293 |
| FT3 | 0.057 |
| FT4 | 0.06 |
| Glucose | 0.205 |
| TC | 0.553 |
| HDL-C | 0.109 |
| LDL-C | 0.38 |
| TG | 0.11 |
| BMI | 0.035 |
| SBP | 0.554 |
| DBP | 0.496 |

Figure S1 Estimation of edge difference within the thyroid hormones-metabolic parameters-clinical symptoms network by bootstrapped difference test.


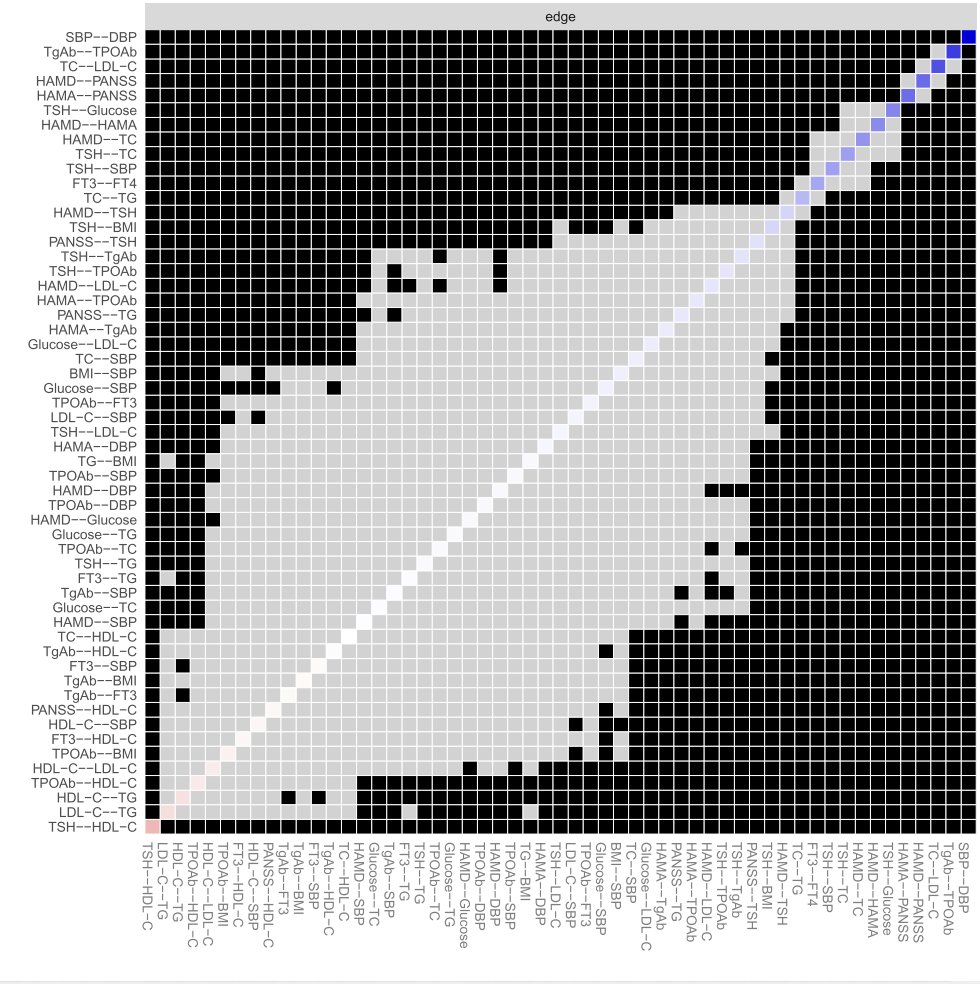


Figure S1. Estimation of edge difference within the network by bootstrapped difference test. Gray boxes indicate edges that do not significantly differ from one another. Black boxes represent edges that differ significantly from one another (α = 0.05).

Figure S2 Estimation of node strength difference within the thyroid hormones-metabolic parameters-clinical symptoms network by bootstrapped difference test.


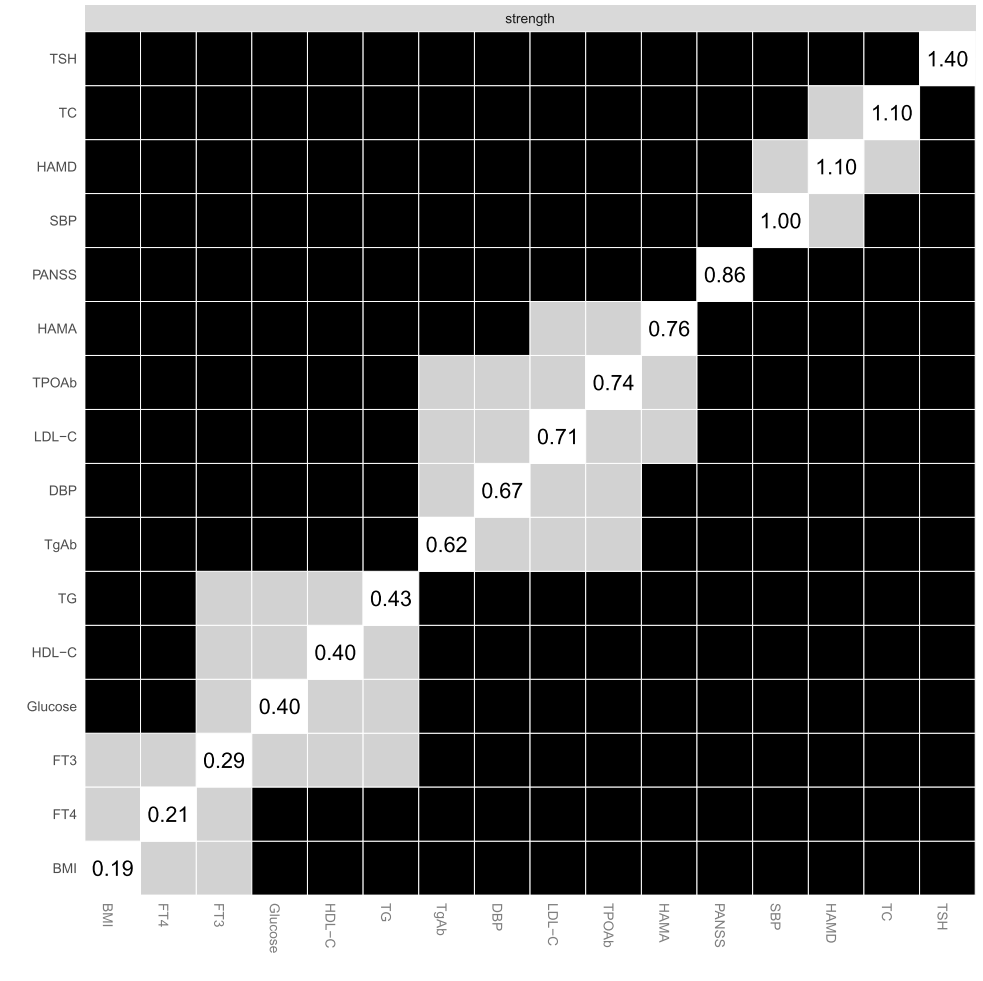


Figure S2 Estimation of node expected difference within the network by bootstrapped difference test. Gray boxes indicate edges that do not significantly differ from one another. Black boxes represent edges that differ significantly from one another (α = 0.05).

Figure S3 The stability of the network


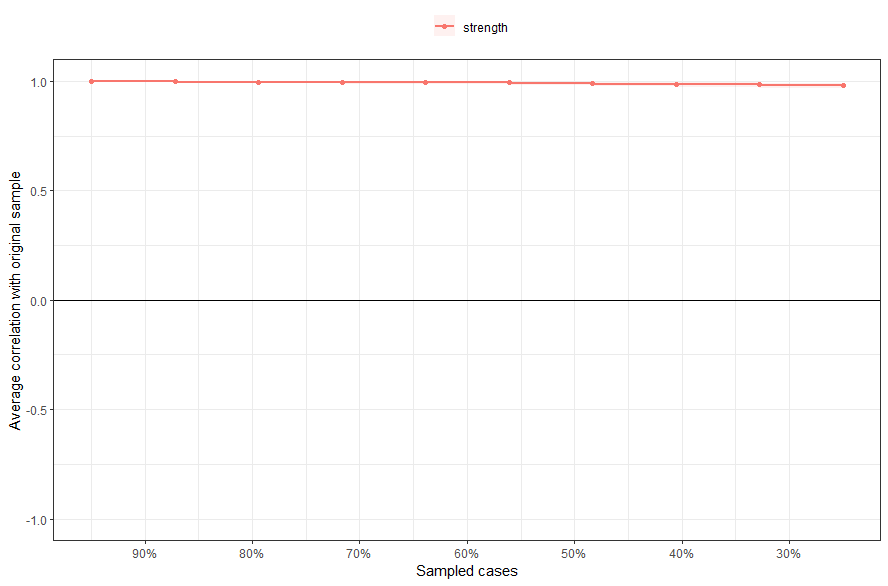


Figure S3 The stability of the network. The stability of node strength by case-dropping bootstrap. The CS-C for the node strength was 0.75

Figure S4 The accuracy of the network


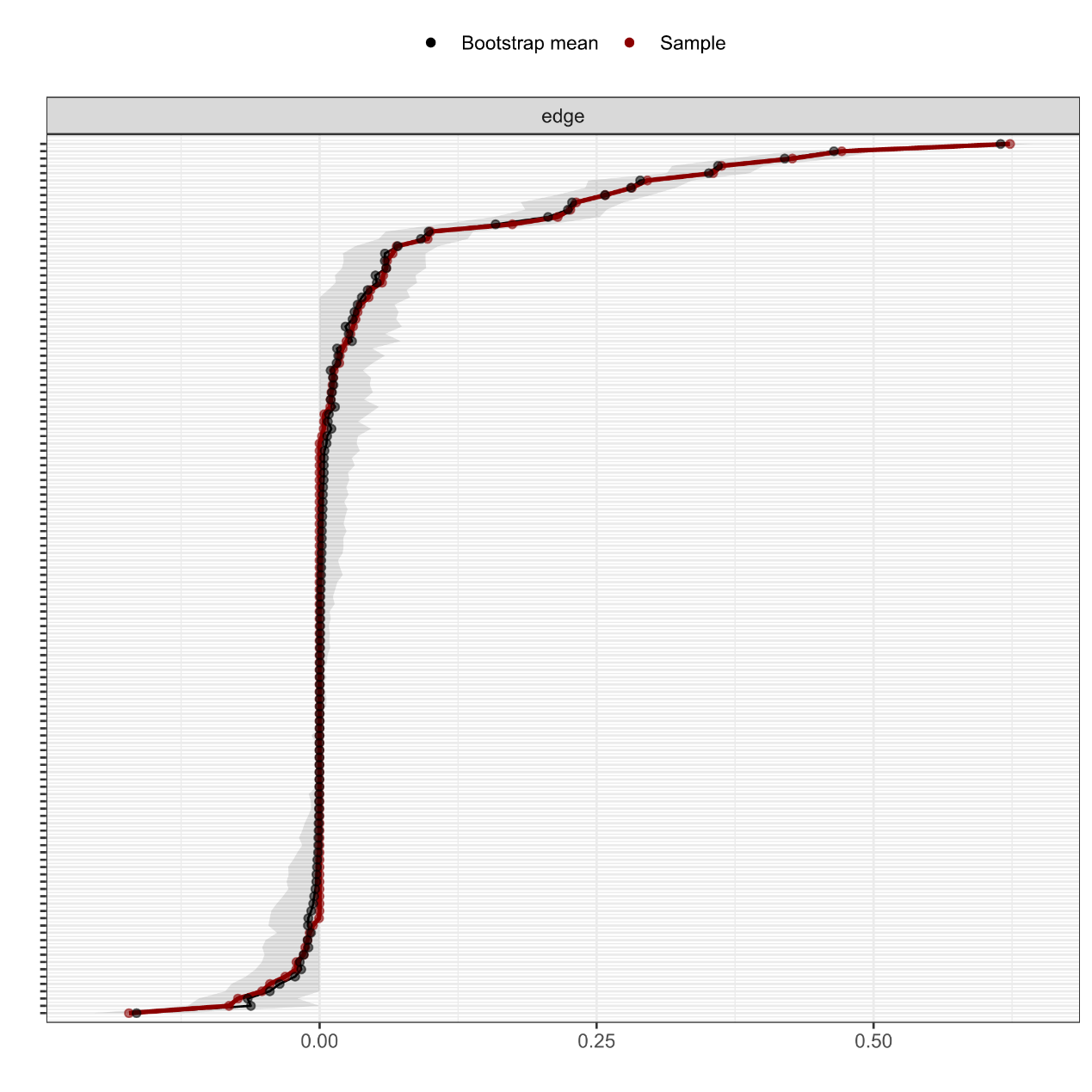


Figure S4. The accuracy of the network edges by non-parametric bootstrapping The grey area represents the bootstrap 95% confidence interval.

-

Figure S5 Network comparison based on gender

1. Network in male


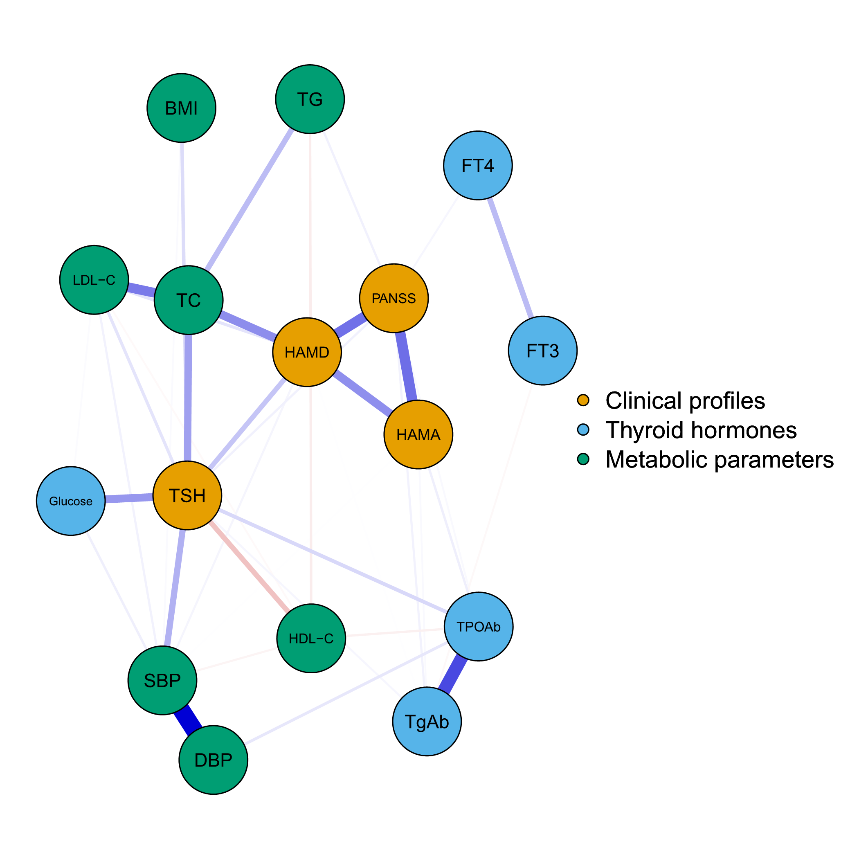


1. Network in female


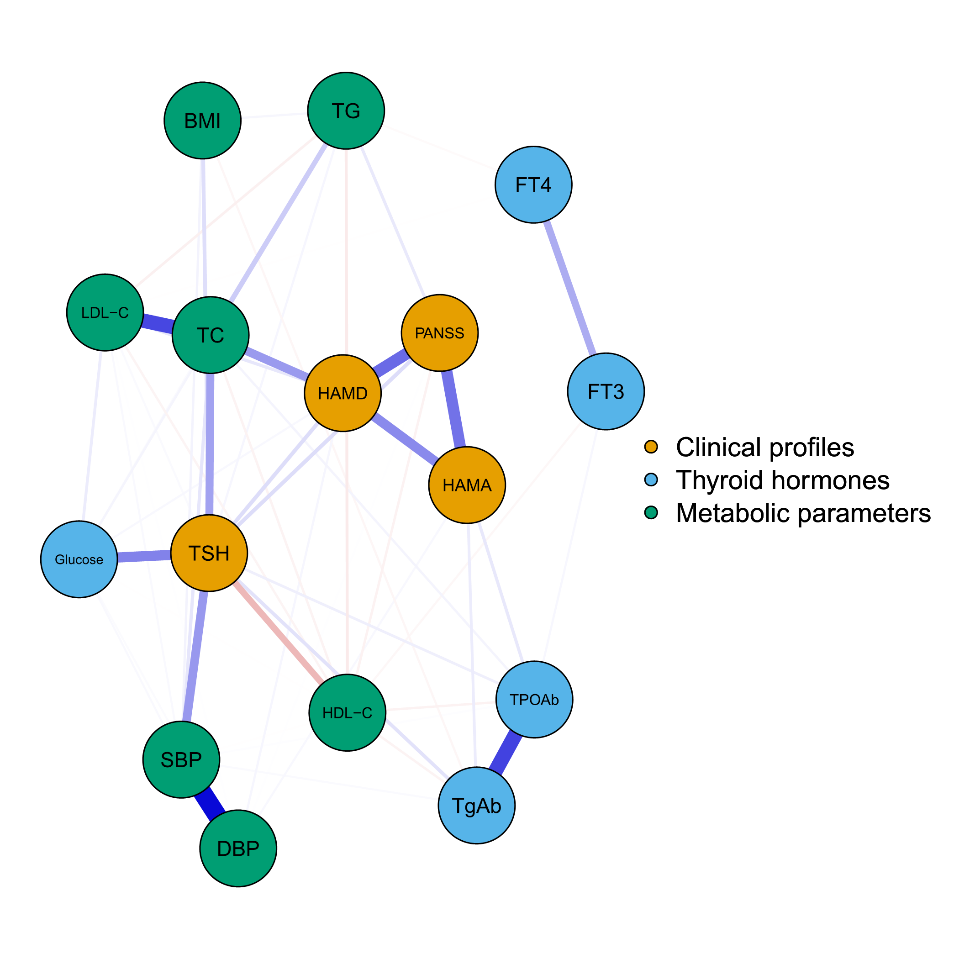


1. Network in young patients with MDD (age 18-45 years old)


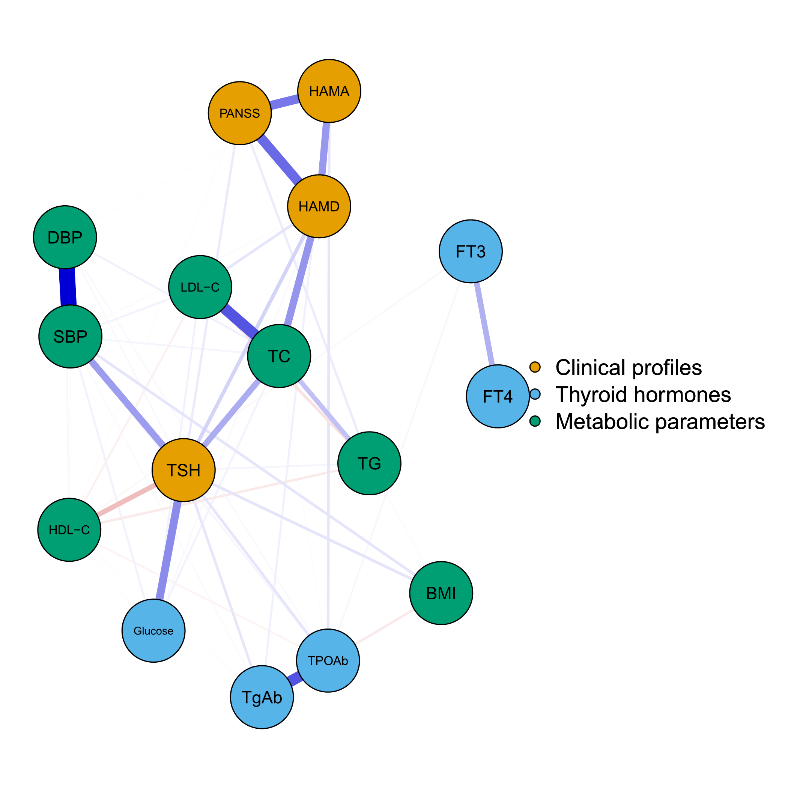


1. Network in middle-aged patients with MDD (age 46-60 years old)


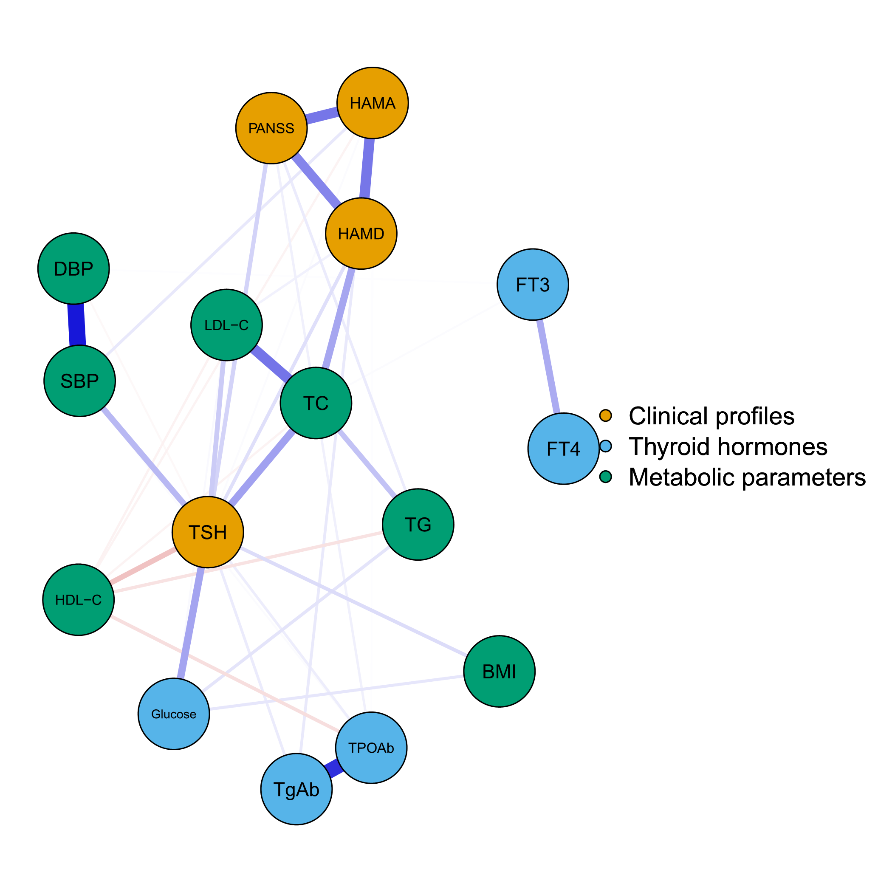


1. Network in patients with long illness duration (illness duration > 5 months)


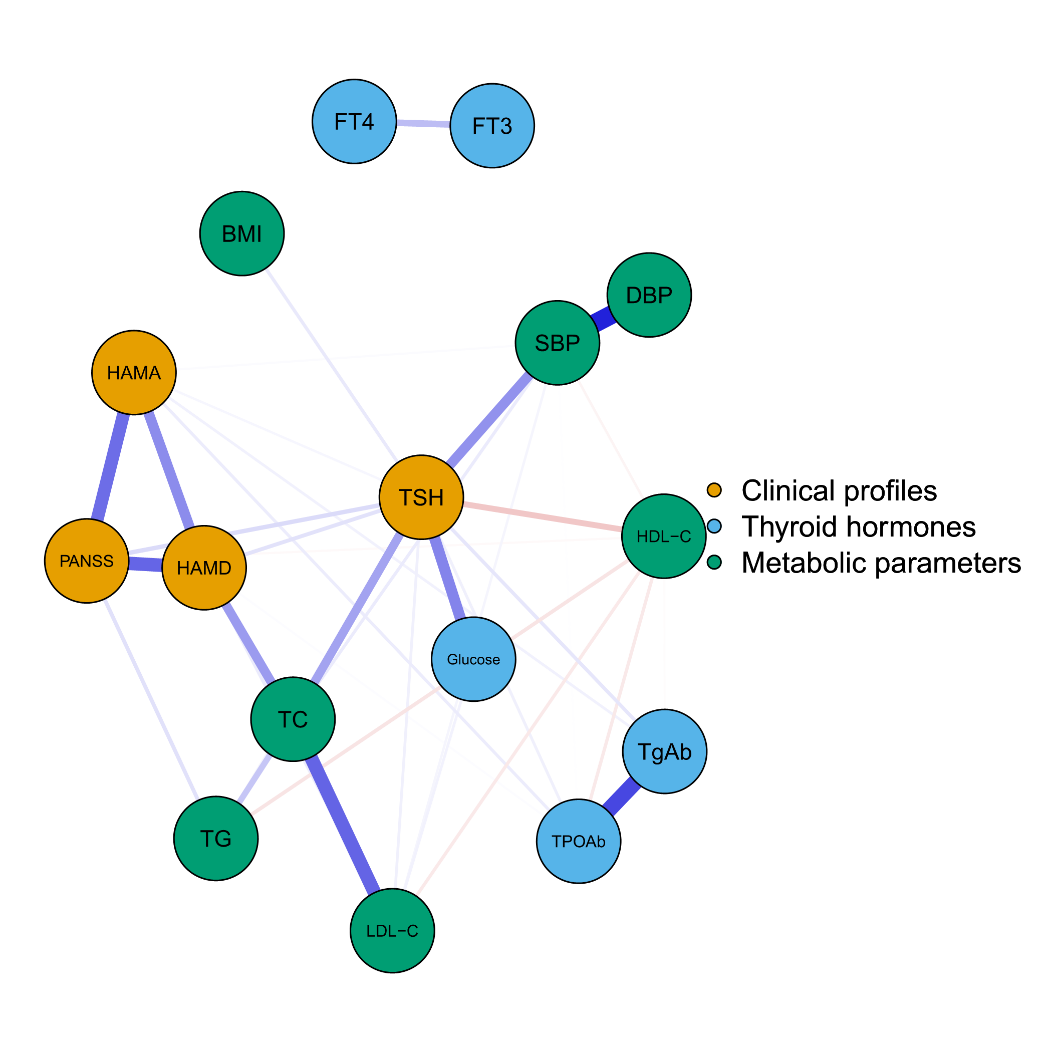


1. Network in patients with short illness duration (illness duration ≤ 5 months)


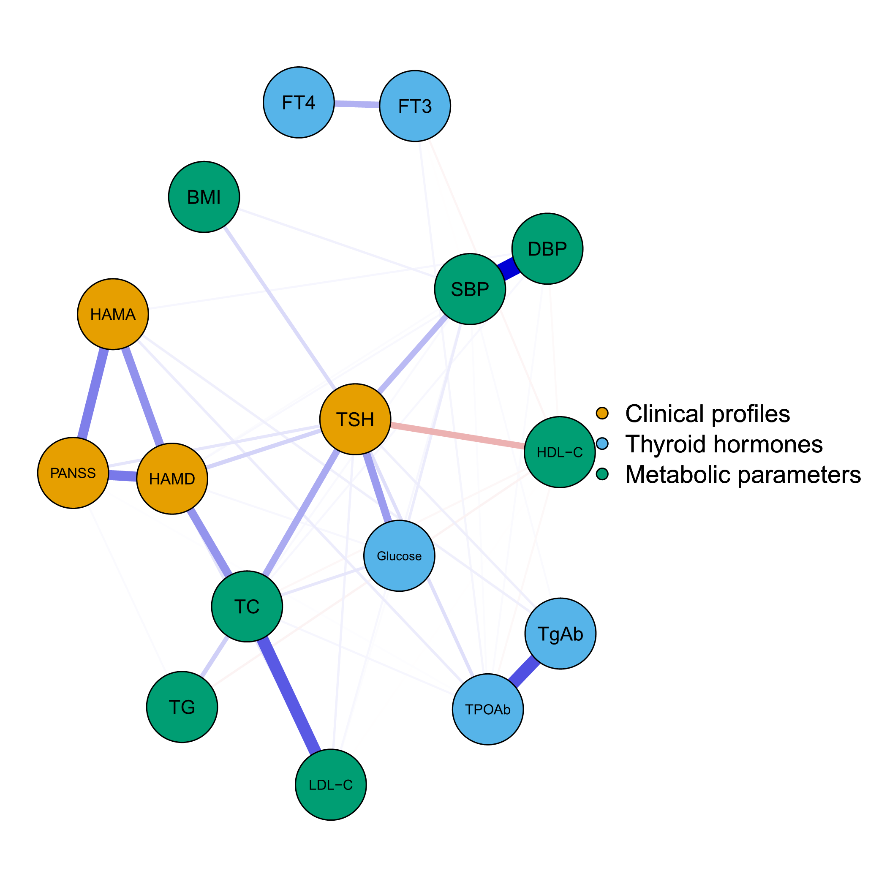


Figure S5 Network comparison by gender, illness duration, and age. The blue, green, and orange nodes represent thyroid hormones, metabolic parameters, and clinical profiles, respectively. Blue edges indicate positive association while red edges indicate negative association. Thicker edge implies a stronger association.
